# Monthly Digest

**Published:** 1889-04

**Authors:** 


### MONTHLY DIGEST.

### The Trend of Dental Thought as Presented by our Contemporaries

*February Southern Dental Journal, Western Journal, International, February and March Reviews, March Archives, Cosmos, Items of Interest, Ohio Journal.)*

### IMPLANTATION.

The subject of implantation is becoming more and more prominent in society discussions and clinics.

The *Cosmos* republishes, from the *Brooklyn Medical Journal*, extracts from a paper by Dr. Rodrigues. The paper is illustrated by drawings made from sections of the maxillæ, cut through the sockets of the teeth, and showing the relative positions of the roots, the nares, the antrum, the dental nerve canal, etc., in a manner not to be found in any of the text-books, but which illustrates points of vital interest to those undertaking the operation of implantation.

Foramina not figured in Gray (nor in any other work seen by Dr. R.,) are found by him to be constant in the bones, both inferior and superior. A knowledge of the location of these foramina is of value in preventing the severe neuralgia liable to follow touching these unsuspected nerves, or nervelets, in drilling a socket. Dr. Rodrigues points out that the labial plate of the alveolus should be left very thin in drilling sockets for superior central incisors, as it is in the normal socket; otherwise the palatal canal may be impinged upon. There is also less danger if space is left between the centrals, working towards the laterals and away from the median line. The central incisor root should not be too long, as there is risk of entering the cavity of the nares.

There is little danger of reaching either antrum or nares, in drilling cuspid sockets, but there is a vascular supply from vessels having no fixed canals, but which penetrate the process immediately over the cuspid, necessitating caution in drilling even in this apparently safe region.

In drilling sockets for bicuspids, the proximity to the floor of the antrum is difficult to determine, the lower portion of the malar process, easily found with the finger, being an approximate guide.

In the inferior maxilla, in the molar region, the dental nerve canal curves forward, and the burring instrument should therefore slant towards the tongue, rather than straight down. In the bicuspid and cuspid region the canal is directly under the teeth. Behind the central incisors Dr. Rodrigues finds, in all specimens, foramina analogous to the anterior palatal in the upper jaw, though not figured in Gray or other works. Another possibility to be borne in mind in drilling in the upper jaw, is that, in tooth sockets filled with new bone, the most canallous portion of the whole bone is often overlaid with a very dense superficial layer, offering great resistance, the drill suddenly plunging into and through the spongy bone beneath, entering the antrum, unless great care is taken.

Dr. G. L. Curtis, in a paper read before the New Jersey State Dental Society (published in the *International*), considers the practical side of this comparatively new field, in which he is experimenting from a scientific point of view, and under the most favorable conditions, with the most gratifying results, as for as a test of from two to three years can constitute success.

Among the principles laid down, Dr. Curtis says: Use only fully developed teeth, and freshly extracted when possible. If dry, they should remain for a few days in a bichloride of mercury solution, 1-5000, lessening the danger of fracturing in cleaning root canals, etc.

When the alveolar wall is quite absorbed, crowns can be attached to very long roots; the palatal root of a molar can be utilized to support a lateral incisor crown. In drilling the socket, care must be taken not to penetrate the antrum, though even this has been done without any evil results being reported.

In all cases the cementum should be completely imbedded.

The canal should be filled through an opening in the masticating or palatal surface, opening through the root wounds the cementum and increases the chance of absorption, which may also result from wounds caused by the use of the forceps.

In the choice of patients, avoid the scrofulous, the syphilitic, pregnant women, or those affected by wasting disease or overtaxed system.

The implanted tooth should be held firmly in place by means of a staple or clasp. Use the cold compress in case of inflammation.

Absorption of the roots of implanted teeth being apparently due to excessive proliferation of new cells, Dr. Curtis suggests the possibility of surrounding the root with some impervious substance, which will be retained in the jaw and hold the crown after the root has been absorbed.

In the discussion of this paper, Dr. E. C. Kirk spoke at length upon the conditions requisite to success. While carefully avoiding any systemic vice, beyond this and underlying it, is the question of the status of the patient's nutrition—whether this is in a condition of equilibrium, or whether the balance is upon the side of repair or that of atrophy. The variable nutritional condition of the individual may account for the variable results, this condition being exceedingly difficult to determine, as we have learned in our treatment of pyorrhœa alveolaris. The trophic centers presiding over certain tissues may be in abnormal condition while the patient is apparently in typical good health.

The method of attachment Dr. Kirk considers to be that of encapsulation.

Dr. Sudduth regards the method of attachment as encapsulation, followed by bony anchylosis, there being no cushion of pericemental membrane between the root of the tooth and the surrounding bone. Dr. Sudduth also spoke of the legal aspect of the question, as to how far the dentist is protected by law in the performance of surgical operations from which disease may possibly follow, asking the question: Are we competent to take charge of such a case, from its medical aspect?

At the clinic of the society, Dr. Curtis implanted a superior central incisor, for Dr. Cornwall, without cocaine. Dr. Cornwall stated that cutting the bone for a socket is felt less than cutting sensitive dentine. The statement having been made that the painlessness of the operation was due to Christian science, or the mind cure, Dr. C. expressed his willingness, for his own satisfaction, to have another vacancy filled without the aid of either Christian science or cocaine.

Dr. Curtis also implanted a second inferior molar for Dr. Seymour, of Asbury Park. From the location, and the recession of the alveolar walls, the operation was difficult, and occupied thirty minutes. A twenty-five per cent. solution of cocaine was used in this case. The tooth was secured to the third molar by a platinum wire staple.

Dr. Smith holds an implanted tooth securely by using, or cutting, a cavity in the tooth and its neighbor and putting in a staple running from one to the other, cemented in and left for two or three months.

Dr. H. C. Herring, Concord, N. C., (Cosmos), says that his only failures in implantation have been with teeth recently extracted. An upper right central, implanted September 26, 1887, and which is now doing good service, and undistinguishable from the other teeth, had been lying in an old chest which had not been opened for seventeen years. Dr. Herring says that he would rather have a tooth implanted twice every year than wear a removable plate. He speaks from personal experience, as a tooth implanted in his own mouth by Dr. E. C. Kirk, came out eight months later, and was immediately replaced by another, the socket being reformed. The second tooth is doing well.

Dr. Herring, (in the January *Southern Dental Journal*), explodes the so-often repeated story of a successful implantation of a porcelain crown upon a leaden root, by Dr. Harris, of Grass Valley,

Cal. The tooth was implanted as described, but proved a failure, dropping out when the ligatures were removed, two or three months later.

Dr. C. N. Pierce reported at the Pennsylvania Odontological Society (*International*) the implantation of a left superior lateral incisor, replacing an artificial lateral which had been worn attached to the cuspid, with its base resting against the gum, and which had produced considerable absorption of the gum and labial process. The apical end of the original root was found in drilling the socket.

At the same meeting Dr. Register reported the implantation of a right superior lateral. This was implanted in the socket of a freshly extracted root, using the natural socket, which, however, had to be deepened somewhat to receive the implanted tooth. There was no soreness on tapping, after the first week, and no pus formation. The gum was not wounded, and the festoon remained perfect.

Dr. J. M. Edmunds, in the *Record* (State Society, New York,) suggests the novel idea of implanting a capsule (made of platinum foil dipped in hydrochloric acid and then in melted lead), in the socket of an extracted tooth, the capsule being molded upon the extracted root. The socket, after syringing with five per cent. carbolic acid, or, if there has been an abscess, 1-6000 bichloride of mercury, is cocoanized with six per cent. hydro-chloride cocaine, by means of a saturated pledget of cotton placed in the socket for from eight to fifteen minutes. The capsule is then inserted and burnished perfectly to the walls of the alveolus. When firmly encysted, a crown is to be attached.

If an artificial socket is made, an impression of the socket is taken by means of modeling composition on a piece of soft wood cut to suit. This model is molded in sand and a zinc cast made, on which the capsule is made as before.

Dr. Edmunds suggests the use of these implanted capsules for the support of entire dentures, upper or lower, implanting one on each side in the posterior part of the arch and one in location of each cuspid. For the denture a rim of gold is to be swaged to fit the gum and four abutments soldered to it to telescope in the implanted capsules. Countersunk teeth may be attached to the gold rim with pink rubber, or the rim may be made of platinum for continuous gum work.

### PORCELAIN FILLINGS.

Porcelain as a filling material is gaining ground rapidly, the methods of utilizing this very refractory material being quite numerous.

Inlays ground from pieces of manufactured teeth ; inlays of all sizes and shapes kept in stock cut from the roots of continuous gum teeth, or from cylinders made for this purpose ; inlays baked in a platinum matrix which has been burnished to the walls of the cavity ; tips with pins for restoring corners of incisors or portions of crowns of posterior teeth—are among the forms adapted to various cases and conditions.

In a clinic of the First District Society, New York, reported in the *Cosmos*, Dr. C. H. Land attached two-thirds of the end of a superior central incisor for a lad twelve years old without destroying the pulp. By this method he avoids having the neck of the shell impinge upon the soft tissues, and also a wet joint which is a great advantage. For porcelain fillings Dr. Land burnishes thin platinum into the cavity, as a matrix, in which tooth-body is baked, thus getting such a very close adaptation that by virtue of the laws of adhesion, cement can not wash out. A filling made in this way can be inserted in five minutes, a great advantage, especially in working for children.

Dr. B. C. Russell (*Ohio Journal*) uses inlay-cylinders clamping one end securely to the porte-polisher and grinding till it exactly fits the cavity, which is made circular, with straight sides and flat bottom. Marking the depth of its insertion with a fine-pointed pencil, a deep groove is cut with a thin corundum disk ; the inlay end is then smeared with oxyphosphate cement, inserted in the cavity and with a little wrench the cylinder is snapped off. When the cement has hardened the inlay is ground and polished flush with the tooth. The advantage of the cylinder is the firm grip with which it is held by the porte-polisher.

Dr. A. H. Thompson (*Review*) uses sections ground from the gum-colored portion of manufactured teeth as inlays in the labial aspect of the necks of the anterior teeth, restoring the appearance of the receeded gums. If the cavity is large two pieces are used fitted together at an angle in the centre like the comb of a roof, to restore the fullness of the gum.

Dr. C. Thomas, Chicago Dental Society, uses the platinum matrix—making fillings for all cavities. If for restoring crowns he inserts a pin, or two pins, in the filling, cutting retaining points in the cavity for the reception of the pins. The porcelain is ground to the proper contour outline after the cement has hardened.

Dr. E. M. S. Fernandez uses the English tips which are made of clear porcelain similar to tooth structure.

Dr. W. X. Sudduth suggests grinding the porcelain filling into the tooth putting emery in the cavity, thus securing a perfect adaptation.

Dr. Prosser, St. Louis Dental Society, reported in *Archives*, has not found porcelain inlays lasting or satisfactory in appearance, and would use them only in exceptional cases; considering a well-finished gold filling quite as sightly and more durable.

Dr. Wm. Conrad raises the objection to Dr. How's inlays that the cavity is made to fit the inlay, necessitating too great destruction of sound tooth bone to entitle the method to the claim of conservative dentistry. He also considers their retention as very insecure, gold being too conspicuous; cements working out; gutta-percha not filling the interstices.

Dr. J. B. Newby also looks upon their retention very unsafe and requiring constant attention.

Dr. Morrison would use sections of English tooth, or some kind of shell which can be ground and polished.

### COCAINE.

Everywhere the note of alarm is being sounded in regard to the use of cocaine. The paper read by Dr. Pruyn, at the recent anniversary meeting of the Chicago Dental Society, together with the discussion of the same sum up quite fully the dangers that may follow its injudicious use, the physiological and the toxicological effects, the contra-indications, and the antidotes. This may be read in full in the *Review*, our abstract, however, giving the most important points.

In the New Jersey Dental Society, reported in the *International*, Dr. Dwinelle said that he wished to sound a note of alarm in regard to its use. He spoke of idiosyncracies of constitution, the best possible safeguard being *a very limited limit* to the quantity used—say one-eighth grain as used by Dr. Kirk.

Dr. Kirk thinks its physiological action has been pretty well settled—that of a vaso-motor constrictor, the antidote being nitrate of amyl, one of the most potent remedies against syncope or heart failure. The exhibition of ether or of ammonia either by hypodermic injections or by the stomach acts also as heart stimulants.

Dr. Curtis said that he believes we can very readily get a toxic effect, though he has not seen any really bad effects from its use. He never gives it except when demanded by the patient as he thinks a person is better qualified to stand pain without the drug than with it.

Dr. Ottolengui (*Cosmos*) read before the First District Dental Society, New York, a paper of the toxic effect of cocaine, giving a compilation of the replies received to a card published some time since in the *Cosmos* requesting reports of cases. The cases reported are very interesting and show an alarming frequency of toxic effects. A summing up of the discussion of the paper in the society points to the facts that while the toxic symptoms are very alarming the contra-indications are also very positive and the antidotes well-known, powerful and reliable, so that the drug may be used with propriety and safety where its use is not contra-indicated, provided that the antidotes are at hand.

M. Moreau in the *British Journal of Dental Science*, (quoted in the *Southern Dental Journal*) gives his personal experience in the hypodermic injection of cocaine. Being about to undergo a long and very painful operation on some of his teeth he gave himself a hypodermic injection of one-third grain in the right forearm, the affected area being also painted over; the ten per cent. solution was also injected in the cavity of a molar—in all, about one and one-third grains being thrown into the tissues. From his experience of disturbance of sight, convulsive, trembling of the upper extremities, and other "accidents of a very serious nature," lasting over an hour and-a-half, he says he certainly should never advise anyone to try this method of anæsthesia.

Mr. Labordi also says death might result from syncope due exclusively to cocaine.

Dr. Geo. S. Staples (*Archives*) uses as a local anæsthetic twenty grains cocaine dissolved in one ounce concentrated ether to which is added one ounce pure oil of peppermint. Apply for from six to twelve minutes in the extraction of sore teeth when the gums are inflamed.

### ANTISEPTICS.

At the December meeting of the Pennsylvania Odontological Society, reported in the *International*, Dr. Jas. Truman expressed the opinion that antiseptics will, without doubt, occupy a leading position in the therapy of the future. The micro-organisms found in the oral cavity must be combatted there before they enter the general circulation. He thinks there would be little to fear from contagion through the atmosphere if *the mouth* were kept in a sterilized condition, especially if the antiseptic is used in the form of a spray as recommended by Dr. Register.

Dr. Register spoke of the great advantage of the atomizer. The mouth is clean, the teeth are clean, the fillings are clean. Oil of peppermint forms a pleasant antiseptic especially for children who use the atomizer willingly, effectually washing away the gummy substances which cling to the teeth and form a medium for the development of bacteria.

Dr. C. N. Peirce also spoke of the great value of antiseptic mouthwashes in modifying the progress of caries and preserving the teeth.

Dr. Kirk commends listerine as an agreeable antiseptic mouth-wash.

Dr. Sudduth ranks bichloride of mercury highest for root canals; carbolic acid as a stimulant antiseptic. For daily use in the mouth hydro-naphthol and silica fluoride of soda with combinations of zinc and bismuth have been recommended. Oil of peppermint, wintergreen, or cinnamon are good antiseptics for use in mouth washes.

### SULPHO-CARBOLIC ACID.

Laplace has found that a mixture of twenty-five per cent. crude carbolic acid with an equal quantity of concentrated crude sulphuric acid gives a thick, syrupy, dark-drown mass which possesses great disinfectant power, inferior only to a five per cent. carbolic solution or a one one-thousandth acid solution of bichloride of mercury. No equally cheap, attainable, and effective disinfectant is known. (*International.*)

### BENZOIC-SULPHIDE OF SODIUM OR "NATRIUMSULFIBENZOAT"

Is according to the experiments of Heckel and other German surgeons a valuable antiseptic equal to carbolic acid, and superior to both sublimate and iodoform, being perfectly harmless even when given in large doses and also free from any disagreeable features. (*International.*)

### OXYCYANIDE OF MERCURY

Is less irritant than solutions of sublimate and there is also less absorption by the tissues. For these reasons it gives better results on suppurating surfaces as in rendering a mucous surface antiseptic. Tested by its power of keeping soup its antiseptic power is six times greater than the bichloride of mercury. (*Archives.*)

Dr. Dujardin-Beaumetz, quoted in the *Ohio Journal*, says, to get the maximum of disinfectant power from carbolic acid add to every one hundred parts of water two parts each of carbolic and tartaric acid.

---

Cocaine in Quinsy.—The *British Medical Journal* of May 19, 1888, contains an article by Dr. de Havilland Hall in the treatment of acute tonsillitis by cocaine. He reports several cases in which the disease had been cut short by the free application to the fauces of a twenty per cent. solution of cocaine, and believes that the drug acts by diminishing the sensibility so that deglutition can take place without pain, and also by diminishing the local congestion so that the inflammatory process is arrested. It would appear that cocaine is more active after the throat has been cleansed by a solution of bicarbonate of soda.—*Sacramento Medical Times.*

